# Geographical Representation of Low- and Middle-Income Countries in Randomized Clinical Trials for COVID-19

**DOI:** 10.1001/jamanetworkopen.2022.0444

**Published:** 2022-02-25

**Authors:** Mahesh Ramanan, Steven Y. C. Tong, Aashish Kumar, Balasubramanian Venkatesh

**Affiliations:** 1 Critical Care Division, The George Institute for Global Health, University of New South Wales, Sydney, Australia; 2 Department of Infectious Diseases, The University of Melbourne at the Peter Doherty Institute for Infection and Immunity, Melbourne, Australia; 3 Logan Hospital, Meadowbrook, Australia

## Abstract

This systematic review examines geographical representation of low- and middle-income countries in randomized clinical trials for COVID-19 compared with high-income countries.

## Introduction

COVID-19 has had a major impact on all countries and regions globally. The populations where randomized clinical trials (RCTs) have been conducted may not reflect the burden of disease.^1,2^

## Methods

In this systematic review, we evaluated the proportion of participants recruited in RCTs for COVID-19 from low- and middle-income (LMIC) countries compared with high-income countries (HIC) (using World Bank definitions) relative to disease burden in these regions. We also examined these proportions stratified by COVID-19 vaccine and nonvaccine RCTs. We followed the Preferred Reporting Items for Systematic Reviews and Meta-analyses (PRISMA) reporting guideline.

Using PubMed and manually searching, we identified COVID-19–related trials published from January 1, 2020, to June 6, 2021, in the 6 highest ranking internal medicine journals: *New England Journal of Medicine*, *Lancet*, *JAMA*, *Annals of Internal Medicine*, *JAMA Internal Medicine*, and *BMJ* (eMethods in the Supplement). A study flow diagram is provided (eFigure in the Supplement). Data regarding trial sponsor, recruitment by country and other trial characteristics were extracted. Authors were contacted where recruitment by country was not publicly available. COVID-19 case data were obtained from the World Health Organization COVID-19 Dashboard on July 18, 2021. Statistical analysis was conducted using Stata version 17.0 (StataCorp) and Excel (Microsoft Corp) from July 13 to July 17, 2021.

## Results

There were 295 845 participants in 71 RCTs (75% nonvaccine and 25% vaccine), of which 247 631 (84%) were recruited in HIC-sponsored RCTs and 48 214 (16%) in LMIC-sponsored RCTs. Forty-nine RCTs (69%) had a HIC sponsor (Table).

**Table.** Characteristics of Included Studies

Characteristic	Studies, No. (%) (N = 71)
Income status of sponsor country	
High income	49 (69)
Low-middle income	22 (31)
Income status of first author country	
High income	49 (69)
Low-middle income	22 (31)
Income status of last author country	
High income	47 (66)
Low-middle income	24 (34)
Journal of publication	
* New England Journal of Medicine*	30 (42)
* JAMA*	16 (23)
* The Lancet*	14 (20)
* Annals of Internal Medicine*	4 (6)
* JAMA Internal Medicine*	4 (6)
* British Medical Journal*	3 (4)
Intervention type	
Pharmaceutical	50 (70)
Vaccine	18 (25)
Nonpharmacological	3 (4)
Participant recruitment	
Mean (SD)	4167 (9396)
Median (IQR) [range]	479 (228-2265) [25-43 783]
Total	295 854
Recruitment by HIC vs LMIC available^a^	284 509
Recruitment by country available^b^	282 464

Abbreviations: HIC, high-income countries; LMIC, low-middle income country.

^a^ Data available for whether participants were from high income or low-middle income countries.

^b^ Data available for specific country of recruitment of participants.

The majority of participants were recruited from HICs (223 666 of 284 509 [79%]). Of the 58 798 (21%) recruited in LMICs, none were from low-income countries, 1074 (2%) were from lower-middle income countries, and 57 724 (98%) were from upper-middle income countries (UMIC). Recruitment by specific country was available for 65 RCTs (92%), which recruited 282 464 (95%) participants; 223 666 participants (79%) were from 23 HICs, and 58 798 (21%) participants were from 11 LMICs.

Vaccine RCTs involved more participants (203 274 of 295 854 [69%]) than nonvaccine RCTs (92 571 of 295 854 [31%]). HICs recruited 150 413 participants (74%) in vaccine RCTs with the remainder from UMICs (52 456 participant [26%]). Nonvaccine RCTs recruited 79 566 participants; 73 253 (92%) from HICs and 6313 (8%) from LMICs (of which 1074 [17%] were from lower-middle income countries and 5239 [83%] from UMICs).

The Figure shows the ratio of participants to COVID-19 disease burden by country income level. Although countries with greater reported number of cases contributed more trial participants, the number of participants from HICs (eg, the United States [101 631 patients] and United Kingdom [70 433 patients]) clearly outweighed those from LMICs with a similar case load (eg, Brazil [22 920 patients] and India [464 patients]).

**Figure.**
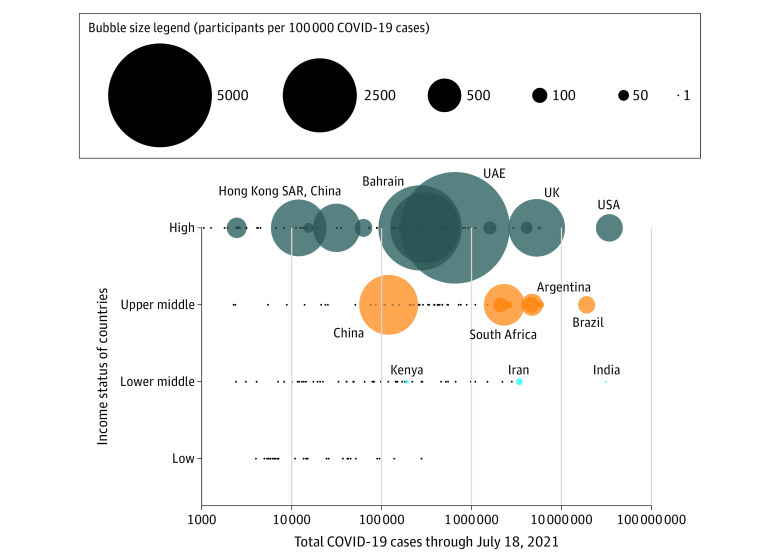
Ratio of Participant Recruitment to COVID-19 Cases by Country and Income Status in Relation to Total COVID-19 Cases Bubbleplot showing participant recruitment to COVID-19 cases ratio stratified by income status and COVID-19 burden. Each bubble represents one country, with countries color-coded by income status. The size of each bubble is proportional to the ratio of participant recruitment to COVID-19 cases in that country. A larger bubble therefore means that that country has recruited a larger number of participants into trials relative to the total number of COVID-19 cases in that country. Overall, there were 80 high-income countries, 56 upper-middle-income countries, 55 lower-middle countries, and 27 low-income countries, of which 23 high-income, 8 upper-middle-income, 3 lower-middle-income, and 0 low-income countries recruited patients. The remaining countries with no participants recruited in COVID-19 trials are represented as small black circles.

## Discussion

COVID-19 has amplified inequalities in global health and socioeconomic outcomes between HICs and LMICs.^2,3^ These disparities extend to both participation in and leadership of COVID-19 RCTs published in leading medical journals. The majority of trial participants and sponsors were from HICs reflecting available research infrastructure and opportunities for sponsorship. Our findings have broad implications for global generalizability of results from these RCTs to LMIC populations, prioritization of research questions most relevant to LMIC, and for building research infrastructure in LMICs to perform high-quality RCTs.^4^

Limitations of this study include our consideration of RCTs published in 6 high-ranked journals only. However, these are the studies most likely to influence practice. The official caseload is highly likely to substantially underestimate true numbers, particularly in LMICs, and thus disparities between recruitment numbers and caseload should be regarded as a minimum estimate.

The potential role of global health inequalities and their adverse impact on COVID-19 outcomes in LMICs was recognized in the early phases of the pandemic.^5^ The results from our study suggest the need for strengthening research capacity in LMICs, mobilizing funding from diverse sources, identifying relevant translatable research priorities, and establishing research partnerships to mitigate the effect of inequalities on COVID-19 outcomes in LMICs.^6^ Given the importance of vaccine strategies, it is striking that no vaccine trial participants were from LMICs. LMIC participation in COVID-19 RCTs should be monitored and actively encouraged by the global research community as the COVID-19 pandemic continues to unfold.

## Notes

Supplement. **eFigure.** Study Flow Diagram
