# Supplementary material for: Geographical Representation of Low- and Middle-Income Countries in Randomized Clinical Trials for COVID-19
Source: JAMA Netw Open. 2022 Feb 25;5(2):e220444. doi: 10.1001/jamanetworkopen.2022.0444 (PMC8881769; doi:10.1001/jamanetworkopen.2022.0444)
Supplement: Supplement. — eFigure. Study Flow Diagram [file jamanetwopen-e220444-s001.pdf]

Supplemental Online Content

Ramanan M, Tong SYC, Kumar A, Venkatesh B. Geographical representation of low- and middle-income countries in randomized clinical trials for COVID-19. *JAMA Netw Open.* 2022;5(2):e220444. doi:10.1001/jamanetworkopen.2022.0444

eFigure. Study Flow Diagram

This supplemental material has been provided by the authors to give readers additional information about their work.

© 2022 Ramanan M et al. *JAMA Network Open.*

eFigure. Study Flow Diagram

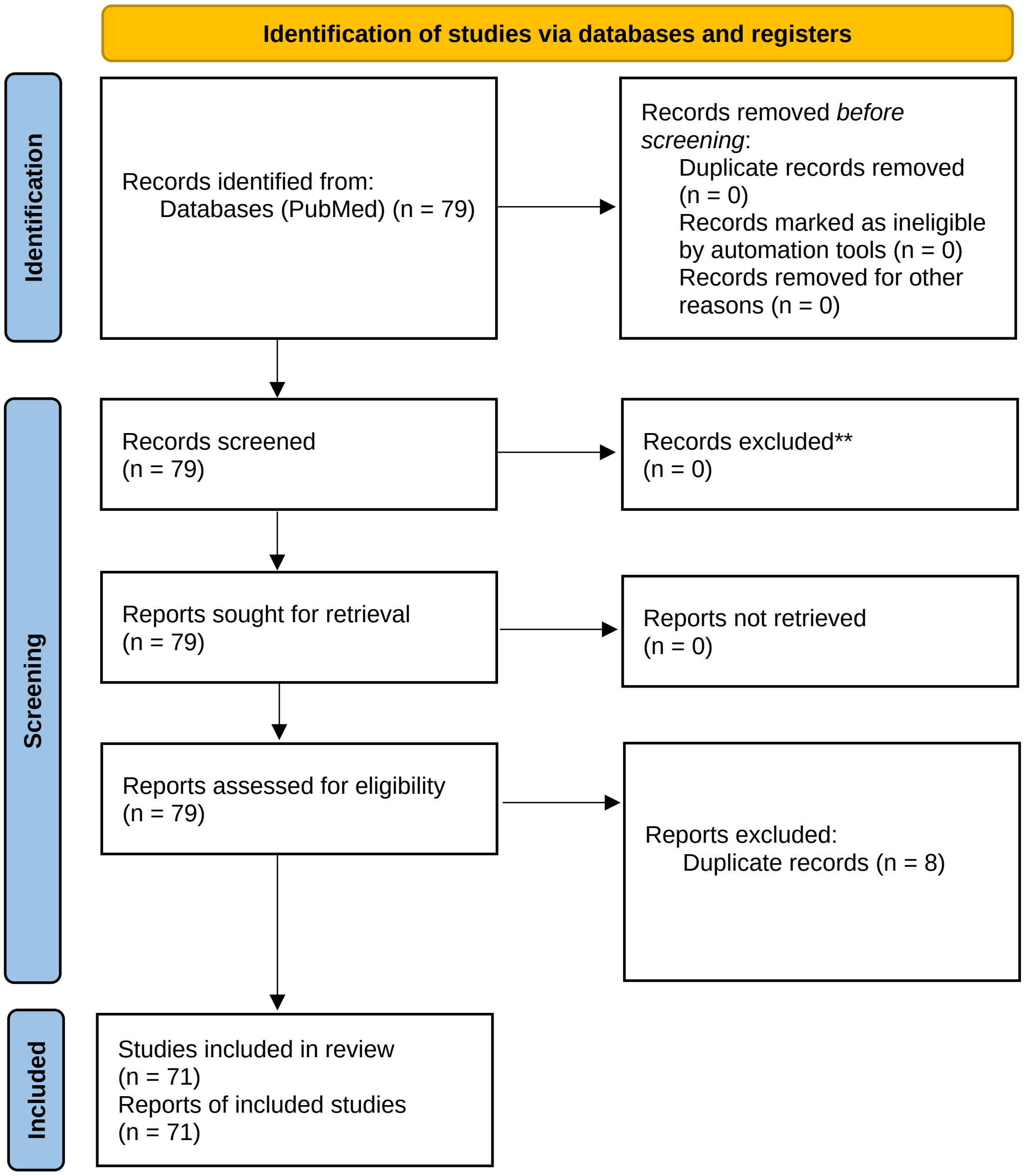

© 2022 Ramanan M et al. *JAMA Network Open.*
